# An uncommon cause of lower gastrointestinal bleeding: a case report

**DOI:** 10.1186/1757-1626-1-235

**Published:** 2008-10-13

**Authors:** Gautam Dutta, Mukta Panda

**Affiliations:** 1 Department of Internal Medicine, University of Tennessee, College of Medicine, Chattanooga, 960 East Third Street, Suite 208, TN 37403, USA

## Abstract

**Background:**

This report calls attention to severe gastrointestinal hemorrhage as a manifestation of small bowel carcinod tumor.

**Patient:**

We report a case of an adult in his 5th decade presenting with massive melena.

**Interventions:**

On presentation the patient was tachycardic (pulse115/min), and hypotensive (BP 80/55 mmHg). His complete physical examination was unremarkable. Rectal exam showed frank blood. Nasogastric lavage produced greenish fluid with no blood. His hemoglobin was 8.1 gm/dl and hematocrit was 25.1%. Panendoscopy revealed an ileal bleeding mass. Interestingly a colonoscopy done 6 months earlier for anemia workup was normal. Emergent laparotomy revealed numerous smaller intraluminal nodules scattered throughout the small bowel.

**Results:**

Histopathology revealed carcinoid tumor.

## Background

Carcinoid tumors are apudomas that arise from enterochromaffin cells throughout the gut. Small bowel carcinoids are most commonly found in the ileum, within 60 cm of the ileocecal valve [1]. The presence of multiple synchronous nodules mandates careful inspection of the entire small intestine to exclude other sites of disease. Grossly, carcinoid tumors appear as firm intramucosal or submucosal nodules, with a yellow cut surface due to their high lipid content. Microscopically, solid nests of uniform small cells with round or oval nuclei are present which show little or no cellular pleomorphism, hyperchromasia, or increased mitotic activity [2]. Because of their indolent growth, most small bowel carcinoids are asymptomatic at presentation and found incidentally. In symptomatic patients, abdominal pain is the most common initial symptom. The pain is most often vague and nonspecific. Intermittent obstruction occurs in 25 percent of all small intestinal carcinoids. Obstruction may be caused by intraluminal tumor, but often results from mesenteric kinking and distortion brought on by tumor invasion and a secondary desmoplastic response [2-4]. Carcinoid syndrome occurs in approximately 10 percent of intestinal tumors, usually in an advanced metastatic stage [2,3]. While rectal carcinoid tumors can present with bleeding, massive bleeding is not a usual feature of carcinoid tumor [1].

Any bleeding distal to ligament of Treitz is considered as lower gastrointestinal bleeding. Most cases of lower gastrointestinal bleeding involve the colon (85%), approximately 10% are actually due to upper gastrointestinal bleeding and only 3% to 5% cases are secondary to bleeding from small intestine [1,2]. While rectal carcinoids may represent more often with lower gastrointestinal bleeding (LGI), small bowel carcinoids can rarely be a cause of obscure bleeding and extremely rarely would cause massive lower gastrointestinal bleeding. A literature search, using PubMed (March 31^st^, 2008) reveals only three cases reported for LGI bleed caused by small bowel carcinoid tumor [5-9]. To our knowledge it appears our case would be the first such to be reported from North America that has been histologically confirmed. Despite its rarity small bowel carcinoids should always be considered in the differential diagnosis of LGI bleeding because they are highly infiltrating and contrary to popular belief may cause significant morbidity.

## Case report

**Figure 1:**
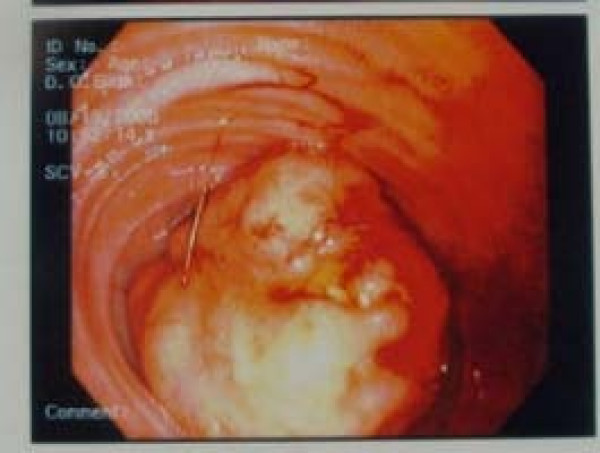
Endoscopic view of the carcinoid tumor.

**Figure 2:**
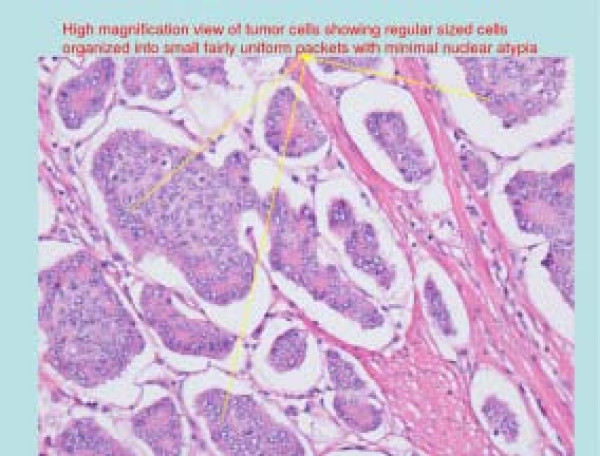
Histopathological view of carcinoid tumor.

**Figure 3:**
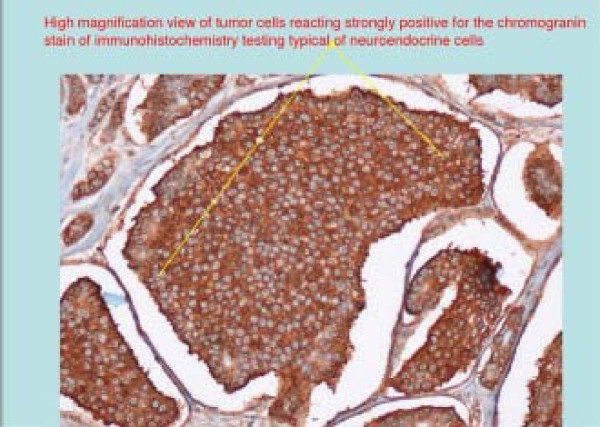
High magnification of carcinoid cells stained positive with chromogranin.

A 44 year old male, presented to the ER with complaints of bleeding per rectum for 1 day. His complaints started with 4 episodes of watery diarrhea with dark blood. He denied any fever, abdominal pain, weight loss, and change in bowel pattern, hematemesis or jaundice. The patient had immigrated to USA 18 months prior from India where he was found to be anemic requiring two blood transfusions over the last four years. Past medical history was significant for anemia only and his home medication was an iron and folate supplement. His anemia was investigated with an esophagogastroduodenoscopy (EGD) and colonoscopy 6 months prior in India, both of which were normal. In the emergency room his vital signs revealed: temperature 98.6°F, pulse 115/min, BP 80/55 mmHg, respiratory rate 32/min and O_2 _saturation 100% on room air. He was not in acute distress, abdomen was soft, non-tender, non-distended, no masses or organomegaly and normal bowel sounds. Rectal exam revealed frank blood. Significant initial lab findings included a hemoglobin level of 8.1 gm/dl and hematocrit of 25.1%. Aggressive fluid replacement via two wide bore IV lines was instituted accompanied by two units of PRBC and he was admitted to the intensive care unit. After stabilization, an EGD did not show any bleeding source. Colonoscopy showed a bleeding mass [Figure 1] in the ileum 20 cm from the ileocecal valve. Emergent laparotomy and excision of the mass was done. Numerous smaller intraluminal nodules were scattered throughout the small bowel. Histopathology of the mass revealed carcinoid tumor [Figure 2, 3]. CT scan of thorax, abdomen and pelvis suggested probable metastasis to lymph nodes [Table 1] without liver involvement. The patient's 24 hr urine 5-HIAA level was 13.1 which is insignificant considering the fact that the patient was getting lorazepam, β-blocker and fruits in his diet, all of which can contribute to a slightly increased level. A 5-HIAA level of > 25 mg/24 hr in the absence of any confounding factors would be a significant level in carcinoid syndrome [10].

**Table 1:** Causes of lower gastrointestinal bleeding (20)

Vascular lesions	Tumors of small intestine	Less common causes
• Angiodysplasia	• Leiomyoma	• Ulcerations of small intestine (Crohn's disease, Meckel's diverticulum, Zollinger-Ellison Syndrome)
• Telangiectasia	• Leimyosarcoma	• Vasculitis
• Venous ectasia	• Carcinoid tumors	• Infectious bowel disease
• Hemangioma	• Adenocarcinoma	• Radiation enteritis
• Arteriovenous malformation	• Lymphoma	
• Dieulafoy's lesion	• Adenomatous polyps	
	• Metastases	

## Discussion

The etiology of massive LGI bleed includes diverticulosis, angiodysplasia, polyps, dieulafoy's lesion, infectious or inflammatory bowel disease [Table 1]. Carcinoid tumors are rare but most common GI neuroendocrine tumors. Small intestinal carcinoids are usually in the ileum and often multiple and rarely present with lower GI bleeding. A literature search using the search engines PubMed & Ovid reveals only three cases reported for LGI bleed caused by small bowel carcinoid tumor [5-10]. Small bowel carcinoid tumors usually present in the sixth or seventh decade of life with abdominal pain or obstruction [1-4]. Symptoms of carcinoid syndrome i.e. flushing, diarrhea are infrequent; occur in less than 10% of patients [3].

Our patient was unique in that he was younger and did not have any of the typical symptoms. He presented with massive LGI bleed which is unusual for small bowel carcinoid that tends to be slow growing and may remain asymptomatic. Further, as demonstrated in our patient, standard endoscopic examination may miss them. Thus these are difficult to diagnose and as in our patient maybe rarely detected before a surgical procedure. As the carcinoid tumor is highly infiltrating early diagnosis is mandatory. Thus a high index of suspicion and the utilization of other modalities for complete examination of the small bowel such as capsule endoscopy, mesenteric angiography, and radionuclide RBC scan should be considered in patients with LGI hemorrhage in whom EGD and colonoscopy are negative.

## Conclusion

Carcinoid tumors can cause massive lower GI bleeding. Because of a substantial false-negative rate for lesions on initial endoscopy as demonstrated in our patient, and also because of the fact that small bowel carcinoid tumors are highly infiltrating thus mandating early diagnosis; an aggressive approach should be taken.

## Competing interests

The authors declare that they have no competing interests.

## Authors' contributions

GD drafted the abstract and manuscript, participated in the patient management. MP supervised the abstract and manuscript, supervised patient management. Both authors read and approved the final manuscript.

## Consent

Written informed consent was obtained from the patient for publication of this case report and accompanying images. A copy of the written consent is available for review by the Editor-in-Chief of this journal.
